# Presence of circulating Her2-reactive CD8 + T-cells is associated with lower frequencies of myeloid-derived suppressor cells and regulatory T cells, and better survival in older breast cancer patients

**DOI:** 10.1186/s13058-015-0541-z

**Published:** 2015-03-10

**Authors:** Jithendra Kini Bailur, Brigitte Gueckel, Evelyna Derhovanessian, Graham Pawelec

**Affiliations:** Department of Internal Medicine II, Centre for Medical Research, University of Tuebingen, Waldhoernlestr. 22, 72072 Tuebingen, Germany; Radiology Clinic, Diagnostic and Interventional Radiology, University Hospital Tuebingen, Hoppe-Seyler-Straße 3, 72076 Tuebingen, Germany; BioNTech AG, An der Goldgrube 12, 55131 Mainz, Germany

## Abstract

**Introduction:**

Breast cancer is one of the most common cancers among women. Its incidence is increasing in many countries and a higher number of older women are now being diagnosed with the disease. Immune parameters are implicated in disease progression, and the frequencies of both myeloid-derived suppressor cells (MDSCs) and regulatory T cells (Tregs), associated with tumour burden, have been suggested to be indicators of poor prognosis in cases of metastatic breast cancer.

**Methods:**

Here, we have assessed the frequency of peripheral Tregs and MDSCs in relation to *in vitro* T cell responses to Her2 antigen in 40 untreated breast cancer patients 65 to 87 years of age at diagnosis.

**Results:**

The five-year survival rate of patients who mounted a CD8+ T cell response to Her2 peptides and had a lower frequency of Lin^−^CD14^+^HLA-DR^−^MDSCs was 100% compared to only 38% in patients without Her2-reactive CD8+ T cells and with higher frequencies of MDSCs (*P* = 0.03). Patients who lacked a CD8 response to Her2 tended to have higher frequencies of MDSCs. Similarly, patients who lacked a CD8 response to Her2 and had higher frequencies of CD4^+^Foxp3^+^CD127^low^CD25^+^ Tregs had only 50% survival compared to the 100% survival of patients who did mount a CD8 response and had lower frequencies of Tregs (*P* = 0.03). A similar trend was observed for activated (CD4^+^CD45RA^−^Foxp3^hi^) but not resting Tregs (CD4^+^CD45RA^+^FoxP3^+^). This survival advantage was observed in both metastatic and non-metastatic patients.

**Conclusions:**

Our data demonstrate a negative role of both MDSCs and Tregs in the prognosis of breast cancer patients, the mechanism of which might be through dampening favourable CD8+ T cell immune responses to tumour-associated antigens.

**Electronic supplementary material:**

The online version of this article (doi:10.1186/s13058-015-0541-z) contains supplementary material, which is available to authorized users.

## Introduction

Breast cancer is one of the most common cancers among women worldwide, the incidence of which increases with age [1]. Surgery, chemotherapy and radiotherapy remain the mainstays of treatment, but currently there is great interest in exploiting the patient’s own immune system to control cancer. However, immunity in older people tends to be less effective than in the young, a phenomenon termed ‘immunosenescence’ [2]. This could compromise any attempts to exploit the patient’s own immune system to destroy their tumour. Recent results using immunomodulatory antibodies are highly encouraging in melanoma, lung cancer and several other tumour types [3-6] but there is thus far rather little experience in older patients or those with breast cancer. Antibodies directed to cytotoxic T-lymphocyte associated protein 4 (CTLA-4), programmed cell death protein 1 (PD-1) or programmed death ligand 1 (PDL-1) now coming into widespread use are commonly perceived as ‘taking the brakes off’ immunity. However, this strategy will only be effective if an immune response is potentially present. In melanoma, we have shown that the presence of peripheral T cells responding to certain tumour-associated antigens (TAAs) predicts extended survival [7]. Human epidermal growth factor receptor 2 (Her-2) is an important TAA in breast cancer, which can be targeted by antibodies and T cells [8,9]. Although a normal self-protein, it is often overexpressed in breast and ovarian cancers and can thus be selectively targeted by antibody; there are also a number of anti-Her2 therapies in early phases of clinical trials that seek to elicit T cell responses in cancer patients [10]. For successful immunosurveillance and exploitation of such host anti-tumour reactivity in immunotherapy, it is likely important for the patient to be able to neutralize or decrease the activity of immunosuppressive cells, both T regulatory cells (Tregs) and myeloid-derived suppressor cells (MDSCs). These have been implicated in negating anti-tumour activity in several types of cancers [11-13]. Thus, tumour infiltration by Tregs has been associated with poor clinical outcome not only in breast cancer, but several other cancers as well [14-17]. It is also possible to extract clinically relevant information on Tregs by analyzing peripheral blood. For routine and repeated monitoring, a blood test rather than resected tumour or tumour biopsies is required. Analysis of cells in suspension obtained from the blood also allows the application of polychromatic flow cytometry for better characterization of cell types than is possible with immunohistochemistry in tissue sections, and avoids digesting the tissue to obtain infiltrating cells. Depending on the co-expression of additional markers like CD45RA, Tregs can be distinguished into activated and resting (aTregs and rTregs). On stimulation of rTregs, forkhead box P3 (FoxP3) expression is upregulated, leading to the differentiation and proliferation of aTreg. The frequencies and ratios of aTreg and rTreg are known to change in some diseases and can provide additional information on the likely suppressive milieu [18].

The other main suppressive cells, the MDSCs, are a heterogeneous population of immature dendritic cells, macrophages and granulocytes [19-21] exerting suppressive activity by several different mechanisms [22,23]. High levels of MDSCs have been negatively associated with survival in different cancers [24-26] including in one breast cancer study.

As the immune system in older people is generally impaired, this may make them more susceptible to infections, and possibly to cancer. They tend to respond poorly to vaccination against infectious agents such as influenza [27]. The implication would be that any anti-cancer immunomodulatory therapy relying on an intact immune system in older people might be compromised by immunosenescence, due to increased suppression and decreased effector function. Here, we investigated whether measurable immune parameters could be found even in older breast cancer patients that were predictive of survival and might provide insights into anti-cancer mechanisms under the most challenging circumstances. We conclude that the presence of T cell reactivity to Her2, related to lower levels of immunosuppressive Tregs and MDSCs, correlates positively with survival of older patients with breast cancer, despite their potentially immunosenescent state.

## Methods

### Patients

Blood samples from 40 patients were collected at the University Hospital Tübingen (Women’s Clinic) from March to November 2009. Blood was drawn upon first diagnosis, prior to any treatment or surgery. Peripheral blood mononuclear cells (PBMCs) were isolated using standard Ficoll-Hypaque gradient and cryopreserved for experimental purposes. Approval for this study was obtained from the Institutional Ethics Committee of University Clinic Tuebingen (71/2009BO2) and a waiver of informed consent was granted for this study. The patients were at different tumour stages grouped according to tumour size (T), nodal status (N) and metastasis (M) (Table 1).

**Table 1:** **Characteristics of the breast cancer patients**

**Clinicopathological parameters**	**(n = 40)**
Age range (years)	65-87
Median age (years)	75
**Tumour stage**	Number of patients
0	3
1	18
2	6
3	6
4	6
Unknown	1
**Tumour size**	
Tis	2
T0	1
T1	21
T2	8
T3	3
T4	5
**Nodal status**	
No	24
N1	6
N2	4
N3	2
Unknown	4
**Metastasis**	
M0	33
M1	6
Mx	1
**Receptor status**	
Triple-negative	8
ER+	33
ER status unknown	1
PR+	31
PR status unknown	1
Her2: 2+	4
Her2 status unknown	2
**Treatments**	
Adj chemotherapy	3
Adj radiotherapy	29
Adj endocrine therapy	30

ER, estrogen receptor; PR, progesterone receptor; Her2, human epidermal growth factor receptor 2; Adj, adjuvant.

### Detection of tumour-associated antigen-reactive-T-cells

T cell responses to Her2 (extra- and intracellular domains) were measured after a 12-day *in vitro* culture. On Day 0 the PBMCs were thawed and resuspended in X-Vivo 15 supplemented with interleukin (IL)-4 (5 ng/ml: Sandoz, Basel, Switzerland) and IL-7 (5 ng/ml: Sterling-Winthrop, New York, NY, USA). On Day 1, mixtures of overlapping peptides (15-mers with an overlap of 11 amino acids) covering the entire sequence of Her2 (PepMix: JPT Technologies, Berlin, Germany) were added, at a concentration of 1 μg/ml. 1 × 10^6^ cells were used for the analysis of T cell reactivity. IL-2 (40 U/ml: Chiron Behring GmbH, Marburg, Germany) was added on Day 3. On Day 12 cultured T cells were harvested and restimulated (0.4 to 0.5 × 10^6^ cells/well) with Her2 PepMix at a concentration of 1 μg/ml or left unstimulated as a negative control for 12 hours. As a positive control, cells were also stimulated with influenza nucleoprotein (NP) and matrix protein (M1) Pepmixes. Golgi-plug (BD Biosciences, Franklin Lakes, NJ, USA) was added at 1 μl/ml to all cultures. After the incubation period, cells were harvested, washed and incubated with Gamunex™ (Talecris Biotherapeutics, Clayton, NC, USA) and ethidium monoazide (EMA, MoBiTec GmbH, Goettingen, Germany) as a marker for dead cells, followed by fixation and permeabilization with Cytofix/Cytoperm (BD Biosciences). The cells were then stained with the following monoclonal antibodies: CD3-Pacific Orange (Invitrogen, Carlsbad, CA, USA), CD4-Pacific Blue, tumour necrosis factor (TNF)-fluorescein isothiocyanate (FITC), IL-2-Alexa Fluor-700, IL-5-phycoerythrin (PE) (BioLegend, San Diego, CA, USA), CD8-allophycocyanin-indocyanine 7 (APC-Cy7), interferon gamma (IFN-γ)-phycoerythrin-cyanine 7 (PE-Cy7) (BD Biosciences), IL-10-allophycocyanin (APC) (Miltenyi Biotech, Bergisch Gladbach, Germany) and IL-17-peridinin-chlorophyll protein-cyanine 5.5 (PerCP-Cy5.5) (eBioscience, San Diego, CA, USA). Cells were immediately measured using a BD-LSR II flow cytometer using the FACSDiva software (BD Biosciences).

### Phenotypic analysis of Tregs and MDSCs

For characterization of Tregs, PBMCs were incubated first with EMA and Gamunex™, followed by indirect staining for CD3 with a primary CD3 antibody (OKT3 supernatant) and a Pacific Orange-conjugated secondary antibody (Invitrogen). After blocking the non-specific binding of the secondary antibody with mouse serum (Merck Millipore, Darmstadt, Germany), cells were directly stained with CD4-Pacific Blue, CD45RA-Alexa Fluor-700, CD8-peridinin-chlorophyll protein (PerCP), CD279-PerCP-Cy5.5, CD127-Alexa Fluor-647 (BioLegend) and CD25-APC-Cy7 (BD Biosciences). The cells were then fixed and permeabilized using the human FoxP3 kit (BioLegend) and the cells were stained for intracellular FoxP3 using a PE-conjugated antibody (BioLegend) according to the manufacturer’s instructions.

For characterization of MDSCs, PBMCs were stained with a cocktail of lineage (Lin) markers (CD3, CD19, CD56)-Brilliant Violet 605 (BioLegend, BD Biosciences), CD14-Brilliant Violet 711 (BioLegend), CD45-V500, CD15-FITC, HLA-DR PerCP-Cy5.5, CD11b APC-Cy7, CD33 Alexa Fluor-700 (BD Biosciences), and CD124-APC (R&D Systems, Minneapolis, MN, USA). All samples were measured using a BD LSRII (BD Biosciences) immediately after staining.

### Flow cytometry data analysis

Data were analyzed using FlowJo software (Tree Star Inc., Ashland, OR, USA). Initially, the duplicates were removed by using an FSC-area versus FSC-height/width plot. These initial steps were done for all flow cytometry datasets. The viable and CD3+ cells were gated to plot CD4+ and CD8+ cells (FACS plots are shown in Figure S1 in Additional file 1). To detect cytokine-producing cells, the unstimulated (negative) control was compared with the stimulated samples and the response considered positive when at least one cytokine was produced by the stimulated sample, defined as an at least twofold increase in the peptide-stimulated culture compared to the unstimulated negative control, as established as a relevant cutoff in earlier studies in melanoma patients [7].

To analyze the Tregs within viable cells, FoxP3+ cells were gated from total CD4+ cells followed by gating of CD127lo and CD25+ cells. The activated Tregs (CD4^+^CD45RA^−^FoxP3^hi^) and resting Tregs (CD4^+^CD45RA^+^FoxP3^+^) were gated by plotting CD45RA against FoxP3 according to a published model [18]. CD4+ cells were the parental population for the analysis of different Treg subsets (gating strategy shown in Figure S2 in Additional file 2).

To analyze the MDSCs within viable cells, CD45+ cells were gated followed by gating CD14+ cells from the Lin(−) population. The HLA-DR(−) population was gated from the CD14+ population defined as MDSC-1 (Lin^−^CD14^+^HLA-DR^−^). The MDSC-2 population (Lin^−^CD14^+^CD124^+^) comprising CD124+ cells was gated from the CD14+ cells by plotting CD124+ against it. CD45+ cells were considered as the parental population for calculating the frequency of different MDSC subsets (gating strategy shown in Figure S3 in Additional file 3).

### Statistical analysis

To compare independent groups, chi-square tests and Mann-Whitney *U* tests were performed. Kaplan-Meier analysis was performed for the survival estimates. GraphPad Prism 6 was used to perform this analysis (GraphPad Software Inc., San Diego, CA, USA). Multivariate Cox analysis was performed using SPSS version 20.0 (IBM Corp, Armonk, NY, USA). A value of *P* <0.05 was considered statistically significant.

## Results

### T cell responses to Her2

T cell responses to mixtures of Her2 peptides were observed in the majority of older breast cancer patients tested (97%, n = 38). Fluorescence-activated cell sorting (FACS) data from a representative donor are shown in Figure S1 in Additional file 1. Most patients exhibited CD4+ T cell responses to Her2 (32/38, 87%), whereas CD8+ T cell responses were detected in only 18 patients (47%) irrespective of whether they had a CD4+ T cell response. Of all the patients, five had only a CD8+ T cell response to Her2 and no CD4+ T cell response. Because most patients possessed CD4+ T cells responding to Her2, these responses would be unlikely to be associated with longer or shorter survival. Thus, the patients were grouped according to whether they mounted a CD8+ T cell response (irrespective of whether they had a CD4+ T cell response). All patients’ T cells responded to influenza peptides, the positive control (data not shown).

### CD8 response to Her2 correlates with overall survival

**Figure 1:**
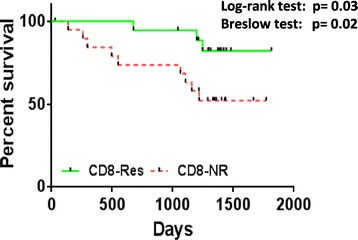
**Survival analysis for CD8-Res vs. CD8-NR to Her2.** Survival analysis for the patients (n = 38) grouped according to CD8+ T cell response to Her2 (CD8-Res) and no CD8+ T cell response to Her2 (CD8-NR). Her2, human epidermal growth factor receptor 2.

Kaplan-Meier survival analysis showed that patients who mounted a CD8+ T cell response against Her2 had a significantly better survival (log-rank test: *P* = 0.03) compared to the patients who did not. There was only 17% mortality in the group of patients with a CD8 T cell response to Her2 when compared to the patients without a CD8+ T cell response, where mortality was 45%. The five-year overall survival rate was 82% for patients with a CD8+ T cell response against Her2 compared with 52% for those without a CD8+ T cell response. Further, having no CD8+ T cell response to Her2 had an early impact on survival as well (Gehan-Breslow test: *P* = 0.02) (Figure 1). Also, five patients who had only a CD8+ T cell response (no CD4+ T cell response) to Her2 had 100% survival compared to the others (data not shown).

When only metastatic patients (n = 6) were considered for Kaplan-Meier analysis according to their response to Her2, those who lacked a CD8+ T cell response (n = 5) had only 25% survival rate compared to the one patient with a CD8+ T cell response still alive at five years (data not shown). Further, the Kaplan-Meier analysis of metastatic and non-metastatic patients showed significantly a better survival for the latter (log rank test: *P* = 0.03) as expected. Also, patients who received adjuvant radiotherapy had better survival (log rank test: *P* = 0.001) compared to those who did not (Table 2). Multivariate Cox analysis was performed for 37 patients considering all significant factors (Model 1). It was observed that having no CD8+ T cell response to Her-2 had an independent negative impact on survival (*P* = 0.014) along with no adjuvant radiotherapy (*P* = 0.001). Next, we performed Cox analysis (Model 2) without considering adjuvant radiotherapy and no association was observed for Her-2 responders (*P* = 0.052) and metastasis status (*P* = 0.12) (Table 3).

**Table 2:** **Survival analysis according to the Kaplan-Meier method**

			**Overall survival rate**		
**Factor**	**N** ^**1**^	**% dead (5 years)**	**%**	**95% CI**	***P***
**Metastasis**					**0.03**
No	33	28	72	0.01-0.8	
Yes	6	50	40	1.1-65.6	
**Estrogen receptor**					0.3
No	9	44	55	0.5-8.1	
Yes	30	23	75	0.1-1.9	
**Progesterone receptor**					0.7
No	12	25	72	0.2-2.9	
Yes	27	29	69	0.3-4.3	
**Her2 status**					0.3
Neg	34	26	72	0.05-2.8	
Pos	4	50	50	0.3-17.9	
**Chemotherapy**					0.2
Yes	3	0	100	0.04-2.3	
No	37	12	65	0.4-21.07	
**Radiotherapy**					**0.003**
Yes	27	19	81	0.03-0.5	
No	13	54	37	1.9-28.6	
**Hormonal therapy**					0.3
Yes	30	27	72	0.1-1.9	
No	10	40	53	0.5-8.2	
**CD8 response to Her2**					**0.03**
Yes	18	17	82	0.09-0.9	
No	20	45	52	1.07-10.4	
**MDSC-1**					0.7
<median	18	28	70	0.2-2.6	
≥median	19	32	66	0.3-4.01	
**MDSC-2**					0.8
<median	18	33	66	0.3-3.7	
≥median	19	26	70	0.2-2.8	

Results of survival analysis according to Kaplan-Meier method and *P* values from Mantex-Cox (log rank) test.

**Table 3:** **Multivariate Cox analysis**

**Model 1**				
**Prognostic factor**	**N** ^**1**^	**% dead**	**Hazard ratio (95% CI)**	***P***
**CD8 response to Her-2**			0.27 (0.07-1.01)	**0.014**
Yes	18	17		
No	20	45		
**Metastasis**			2.76 (0.65-11.64)	0.16
No	32	28		
Yes	6	50		
**Radiotherapy**			0.124 (0.03-0.43)	**0.001**
Yes	27	19		
No	13	54		
**Model 2**				
**Prognostic factor**	**N** ^**1**^	**% dead**	**Hazard ratio (95% CI)**	***P***
**CD8 response to Her2**			0.27 (0.07-1.01)	0.052
Yes	18	17		
No	20	45		
**Metastasis**			2.8 (0.7-10.7)	0.12
No	32	28		
Yes	6	50		

^1^Data for a few patients are missing. Multivariate Cox analysis was performed, where CD8 response to Her2, metastasis and radiotherapy is considered (Model 1), which shows that having no CD8+ T cell response to Her2 has an independent impact on survival (*P* = 0.014) along with patients who did not receive radiotherapy (*P* = 0.001) on five-year survival, whereas, in Model 2 no association was observed for no CD8+ T cell responders to Her2 and metastasis. CI, confidence interval; Her2, human epidermal growth factor receptor 2.

### Associations of tumour characteristics with T cell responses to Her-2

**Figure 2:**
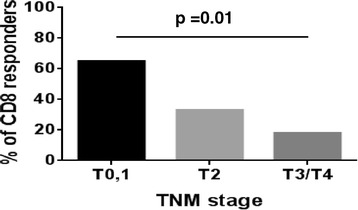
**Distribution of CD8+ T cell responders to Her-2 according to tumour stage.** Percentage of CD8+ T cell responders to Her-2 are significantly higher in patients with tumour stage 0,1 (T 0,1) compared to the patients with tumour stage 3,4 (T 3,4). Her2, human epidermal growth factor receptor 2.

Further, the fractions of patients with CD8+ T cell responses to Her-2 were analyzed for their correlation with different prognostic factors. We found that the proportion of CD8+ T cell responders decreased with increasing tumour stage (Figure 2). There were significantly higher proportions of CD8+ T cell responders in patients with tumours at stage 0 and 1 compared to patients with tumours at stage 3 and 4 (*P* = 0.01). No difference was observed when patients with stage 2 tumours were compared with those with stage 0 and 1 tumours and with those in stage 3 and 4 (Figure 2). There was no difference observed in the percentage of CD8+ T cell responders in patients with tumours with or without estrogen receptor expression, progesterone receptor, or triple-negative breast cancer or metastasis (data not shown).

### Tumour stage and immunosuppressive subsets

We showed above that CD8+ T cell responses to Her2 correlated with tumour stage and patient survival. Thus, we next correlated the frequency of Tregs (n = 30) and MDSCs (n = 37), the two immunosuppressive types, with the tumour stage. For the two MDSC types (MDSC-1 and MDSC-2 as described in the Methods) there was no difference observed between different tumour stages in this cohort. Although there was also no difference observed for the classical Tregs between the tumour stages, a trend was observed for increasing levels of activated Tregs and for the ratio of activated/resting Tregs with increasing tumour stage. There was no difference observed for FoxP3-expressing CD4+ T cells with regard to tumour stage. However, a significant difference was observed for activated Tregs (*P* = 0.04) and for the ratio of activated/resting Tregs (*P* = 0.01) when patients were stratified into one group for tumour stage 0 and 1 and then compared to a second group with tumour stage 2, 3 and 4 (data not shown).

### Overall survival and immunosuppressive subsets

**Figure 3:**
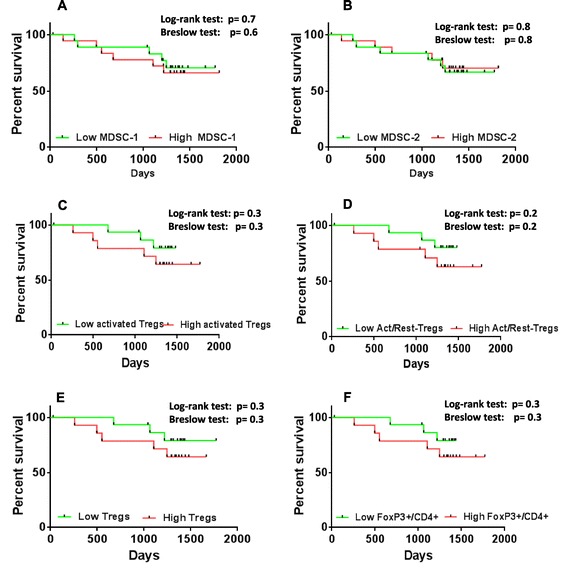
**Survival analysis of immunosuppressive subtypes.** Kaplan-Meier analysis of patients with low (lower than median) versus high (higher than or equal to median) levels of **(A)** MDSC-1 (n = 37), **(B)** MDSC-2 (n = 37), **(C)** activated Tregs (n = 30), **(D)** ratio of activated/resting Tregs (n = 30), **(E)** Tregs (n = 30) and **(F)** CD4+/FoxP3+ T cells (n = 30), showed no significant difference. MDSC, myeloid-derived suppressor cells; Tregs, regulatory T cells.

Kaplan-Meier survival analysis was performed for the immunosuppressive subsets in patients with lower than median and greater than or equal to the median levels of MDSC types (Figure 3A and B) and Tregs (Figure 3C,D,E and F). From the survival analysis it was observed that none of the immunosuppressive subtypes of Tregs and MDSCs correlated with survival, although there was a consistent trend throughout for lower than median levels of Tregs and MDSCs to correlate with better survival in this prospective study.

### Cellular responses to Her2 and different immunosuppressive subsets

Here, we sought to determine if the ability to mount a CD8+ T cell response to Her-2 was associated with the frequency of different immunosuppressive subsets. For this, the patients were grouped according to the presence or absence of CD8+ T cell response to Her-2, and the frequency of Tregs and MDSC types was compared between the two groups.

**Figure 4:**
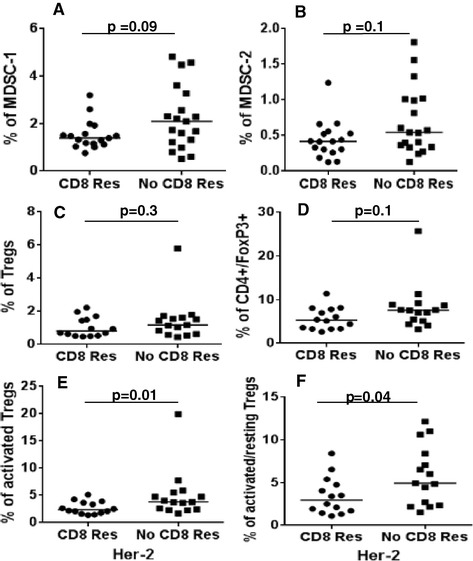
**Percentage of immunosuppressive subtypes in CD8-Res and CD8-NR.** Patients with CD8+ T cell response to Her-2 had low levels of **(A)** MDSC-1 (*P* = 0.09) compared to no CD8+ T cell response. No difference was observed in case of **(B)** MDSC-2, **(C)** Tregs and **(D)** CD4+/FoxP3+ T-cells. The levels of **(E)** activated Tregs and **(F)** ratio of activated/resting Tregs was significantly lower in patients with a CD8+ T cell response to Her-2. Her-2, human epidermal growth factor receptor 2; MDSC, myeloid-derived suppressor cells; Tregs, regulatory T cells.

We found that patients with no CD8+ T cell response to Her-2 tended to have higher proportions of MDSC-1 (*P* = 0.09) but no difference was observed for MDSC-2 (Figure 4A and B). No significant differences were observed for Tregs, resting Tregs (data not shown) or FoxP3-expressing CD4+ T cells (Figure 4C and D). However, patients lacking a CD8+ T cell response to Her-2 had significantly higher proportions of activated Tregs (*P* = 0.01) and the ratio of activated/resting Tregs (*P* = 0.04) was significantly higher as well (Figure 4E and F).

### Survival advantage accrues to patients with a CD8+ T cell response to Her-2 and low levels of immunosuppressive subsets

**Figure 5:**
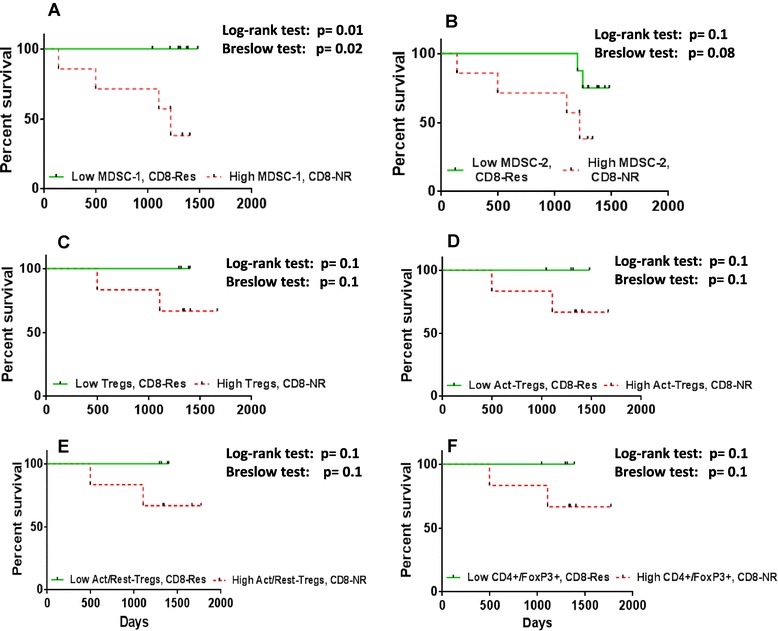
**Survival analysis for patients with CD8-Res and CD8-NR to Her-2 according the levels of immunosuppressive subtypes.** Survival of non-metastatic patients with low levels (lower than median) **(A)** MDSC-1 and CD8+ T cell response to Her-2 had significantly a better survival (*P* = 0.01) compared to the patients with high (higher than or equal to median) levels of MDSC-1 and no CD8+ T cell response to Her-2 (n = 15). No differences were observed in case of **(B)** MDSC-2 for non-metastatic patients (n = 15). Survival for non-metastatic patients with CD8+ T cell response to Her-2 and low (lower than median) levels of **(C)** Tregs (n = 12), **(D)** activated Tregs (n = 12), **(E)** ratio of activated/resting Tregs (n = 12) and **(F)** CD4+/FoxP3+ T cells (n = 12) tended to have a better survival compared to the patients with no CD8+ T cell response to Her-2 and high (higher than or equal to median) levels of regulatory T cell subtypes. Her-2, human epidermal growth factor receptor 2; MDSC, myeloid-derived suppressor cells; Tregs, regulatory T cells.

Kaplan-Meier survival analysis for non-metastatic (tumour stage 0,1,2,3) patients showed that those who mounted a CD8+ T cell response to Her-2 and also possessed low levels (<median) of MDSC-1 had better survival (log-rank test: *P* = 0.01) than patients with no CD8 T cell response to Her-2 and high levels of MDSC-1 (Figure 5A). There was also an early impact on survival of patients with high levels (≥median) of MDSC-1 and without a CD8+ T cell response to Her-2 (Gehan Breslow test: *P* = 0.02) (Figure 5A). Strikingly, the overall survival rate was 100% in patients with low MDSC-1 levels but with CD8+ T cell responses to Her-2 compared to only 38% in patients with high levels of MDSC-1 and no CD8 + T cell response to Her-2. For MDSC-2 type, there was no significant difference observed, although the patients with low levels of MDSC-2 and a CD8+ T cell response to Her-2 had a 75% survival rate compared to 38% in patients with high levels of MDSC-2 and no CD8 T cell response to Her-2 (log-rank test: *P* = 0.1) (Figure 5B). Interestingly, the survival advantage was retained when all patients (metastatic and non-metastatic, data not shown) were considered together, where those with low MDSC-1 levels and CD8+ T cell responses to Her-2 had 100% survival compared to 53% of patients with high levels of MDSCs and no CD8+ T cell response (log-rank test: *P* = 0.03, Gehan Breslow test: *P* = 0.04). For the MDSC-2 subset, there was no significant difference observed for survival (log-rank test: *P* = 0.2) although the patients with low levels of MDSC-2 and CD8 + T cell responses to Her-2 had 75% survival compared to 53% survival in patients with high levels of MDSC-2 and no CD8+ T-cell response to Her-2.

Regarding regulatory T cell subsets, non-metastatic patients with a CD8+ T cell response to Her-2 and low levels of Tregs had an overall survival rate of 100% (Figure 5C. Log-rank test: *P* = 0.1). This was also the case for activated Tregs (Figure 5D. Log-rank test: *P* = 0.1), the activated/resting Treg ratio (Figure 5E. Log-rank test: *P* = 0.1) and the FoxP3+/CD4+ T cell subset ratio (Figure 5F. Log-rank test: *P* = 0.1). Although not statistically significant, this trend has thus been observed for all the subtypes of Tregs. Further, when all the patients (metastatic and non-metastatic, data not shown) were considered together in the Kaplan-Meier analysis, those with low levels of Tregs and with a CD8+ T-cell response to Her-2 had a significantly better survival (Log-rank test: *P* = 0.03) compared to those without a CD8+ T cell response to Her-2 and with high levels of Tregs. Also, a significant early impact on survival was observed in patients with high levels of Tregs and no CD8 T cell responses (Gehan-Breslow test: *P* = 0.03). The overall survival rate was 100% in patients with low levels of Tregs and a CD8+ T cell response to Her-2, compared to 50% in patients with high levels of Tregs and no CD8+ T cell response. Similar results were observed in patients with low levels of activated Tregs (Log-rank test: *P* = 0.06) and FoxP3-expressing CD4+ T cells (Log-rank test: *P* = 0.06) compared to those with no CD8+ T cell response to Her-2 and high levels of activated Tregs and FoxP3-expressing CD4+ T cells. Although patients with a CD8+ T cell response to Her-2 and low levels of resting Tregs tended to have poorer survival compared to patients lacking CD8+ T cell response to Her-2 and with low levels of resting Tregs (*P* = 0.09), patients with a CD8+ T cell response to Her-2 and a low activated/resting Treg ratio did have better survival (Log-rank test: *P* = 0.03, Gehan-Breslow test: *P* = 0.04) than those with no CD8+ T-cell response to Her-2 and a high ratio of activated/resting Tregs. The data were also analyzed by first determining the levels of MDSCs and Tregs and then considering the Her-2 reactive CD8+ T cells. No correlations were observed.

## Discussion

We previously reported that the presence of T cells responding *in vitro* to NY-ESO-1 and/or Melan A peptides was associated with longer survival of late-stage melanoma patients [6]. Melanoma patients tend to be relatively young, so we wished to determine whether these earlier findings were relevant in other tumours mostly occurring in older people whose immune systems may already be compromised by age (‘immunosenescence’). In the present study, we asked whether responses of older breast cancer patients’ T cells to Her-2 peptides were similarly associated with survival and whether the nature of the responding T cells influenced any such association. We found functional circulating Her-2-reactive T cells in 97% of the patients, almost all of whom possessed antigen-reactive CD4+ T cells. However, only about half of these patients also possessed CD8+ Her-2-reactive T cells, and their presence did correlate positively with better five-year overall survival. Interestingly, the survival rate of patients possessing solely CD8+ T cells responsive to Her2 had 100% survival compared to worse survival of those who also had CD4+ T cell responses. This finding emphasizes the benefit of possessing circulating CD8+ anti-TAA cells and implicates CD4+ T cells as potential suppressors of such responses (for example as CD4+ Tregs). Thus, we identified one parameter negatively influencing the proportion of patients with an anti-Her-2 CD8+ T cell response as increasing tumour stage, but another as the presence of suppressive cell types in the blood, both Tregs and MDSCs. An association with MDSCs, but not with Tregs, was also seen in our studies on melanoma [23].

These findings support an important role for Her2-specific T cells in controlling tumour cells *in vivo*. Although most of the patients had tumours with low expression of Her2 (Her2-0,1,2), T cell responses were present in almost all. These results are consistent with studies indicating the benefit of Her2 vaccine in Her2-negative (low expression) patients as well [28-30].

According to the Kaplan-Meier analysis, as expected, metastatic patients had poor survival relative to non-metastatic patients. Nonetheless, this variable did not have any independent impact on survival when considered together with CD8+ T cell responses in Model 1 and Model 2 of the Cox multivariate analysis. However, a CD8+ T cell response to Her2 had an independent positive impact in Model 1 of the Cox analysis. Also, a strong trend was observed in Model 2 for CD8+ T cell responses to Her2.

In our previous study on stage IV melanoma, levels of one of the MDSC subtypes above the median correlated negatively with survival [24], but in the current study we did not observe any direct survival disadvantage for patients with such high levels of MDSCs. This difference might be due to potentially different roles of the various suppressive cell types in different cancers; as mentioned by Montero *et al*. [31] there are different MDSC subtypes that are associated with different type of cancers, in addition to different Treg associations. Nonetheless, when MDSC data were not examined in isolation, but together with other factors, a highly favourable survival advantage (*P* = 0.01) was observed in non-metastatic patients with a CD8+ T cell response to Her2 who also had low levels (lower than median) of MDSC-1 (Lin-CD14 + HLA-DR-). These patients had a 100% survival rate compared to only 38% in patients with no CD8+ T cell response to Her2 and high levels of MDSC-1. These differences were still significant when metastatic patients were included in the analysis as well (*P* = 0.03), with a survival rate of 100% for patients with CD8+ T cell response to Her2 and low levels of MDSC-1 compared to the 53% survival rate in patients with no CD8+ T cell response to Her2 and high levels of MDSC-1. Thus, the prognostic impact of the MDSCs was not limited only to non-metastatic breast cancer.

Earlier studies on breast cancer showed increased levels of one of the MDSC subtypes at higher tumour stage [31], but in the present study, no such differences were observed. This might be because of our small cohort of patients; however, we observed that the percentage of CD8+ T cell responders decreased with increasing tumour stage, consistent with a possible role of immunosuppressive cells in these patients. In line with this, the levels of MDSC-1 were higher in patients with no CD8+ T cell response to Her2, which might indicate the altering capacity and suppressive role played by MDSCs on CD8+ T cells as described in earlier studies [32-34,11,24].

In contrast, the frequencies of Treg subtypes did not correlate with survival, although in other types of cancers like ovarian and renal cell carcinoma, high levels of Tregs have been reported to correlate with poor survival [35,12]. Nonetheless, the non-metastatic patients with a CD8+ T cell response to Her2 and low levels of Tregs, aTregs, ratio of a/r Tregs or FoxP3+/CD4+ T cells showed a strong trend towards better survival: a 100% survival rate was seen for lower levels of all the regulatory T cell subtypes compared to patients with high levels of regulatory T cell subtypes and no CD8+ T cell response to Her2. These survival differences were significant for Tregs (*P* = 0.03) with a survival rate of 100% in patients with low levels of Tregs and a CD8+ T cell response to Her2. Similar results were observed for the ratio of a/rTregs (*P* = 0.03), aTregs (*P* = 0.06) and CD4+/FoxP3+ T cells (*P* = 0.06) when all patients were considered in the analysis. Also, the levels of aTregs and the ratio of a/rTregs were higher in patients with no CD8+ T cell response to Her2, which again suggests a suppressive role played by the regulatory T cell subtypes in this cohort of patients. Previous studies have already shown that increased levels of Tregs do have suppressive effects on T cells in cancer patients [36].

This study emphasizes the clinical relevance of the individual’s circulating CD8+ TAA-reactive T cells, which confer a significant survival advantage. In particular, a combination of reactivity to Her2 antigens and lower circulating suppressive cells translates to better survival of the older breast cancer patient. These findings have several important implications: (1) they emphasize that despite an immune system potentially compromised by immunosenescence, older patients retain the ability to mount clinically relevant anti-cancer responses against Her2, which could be exploited in immunotherapy; (2) they suggest that one mechanism dampening beneficial anti-TAA responses is the presence of suppressive cells, which could be targeted therapeutically to enhance anti-cancer activity.

## Conclusions

The results from the present study indicate that while levels of MDSCs and Tregs alone might not be biomarkers for survival, CD8+ T cell responses to Her2, which do correlate with longer survival, are likely to be affected by these immunosuppressive cells. This may result from the effects of these cells during the analytic *in vitro* culture used here, but also suggests a potentially similar mechanism *in vivo.* To the best of our knowledge, this is the first study on older breast cancer patients showing an advantage of having a CD8+ T cell response to Her2 and at the same time showing the possible negative role played by MDSCs and Tregs. Cancer vaccines targeting Her2 together with the application of strategies to deplete Tregs and MDSCs might thus provide clinical benefit even in older breast cancer patients whose immune systems are generally considered to be compromised by the ageing process.

## Additional files

Additional file 1: Figure S1. CD4+ and CD8+ T-cell response to Her-2. The duplicates were removed by using a FSC-area versus FSC-height/width plot. The viable and CD3+ cells were gated earlier to plot CD4+ and CD8+ cells. A representative plot of control and Her-2 stimulated cytokine (for example: IFN-γ) producing cells for CD4+ T-cells and CD8+ T-cells are shown. Additional file 2: Figure S2. Gating strategy for regulatory T-cell subtypes. The duplicates were removed by using a FSC-area versus FSC-height/width plot. The viable and CD3+ cells were gated to plot CD4+ and CD8+ cells. FoxP3+ cells were gated from total CD4+ cells followed by gating of CD127lo and CD25+ cells. The activated Tregs (CD4^+^CD45^−^FoxP3^hi^) and resting Tregs (CD4^+^CD45RA^+^FoxP3^+^) were gated by plotting CD45RA against FoxP3+ cells. Also, FoxP3+ cells were gated from CD4+ T-cells (CD4 + FoxP3+). Additional file 3: Figure S3. Gating strategy for MDSC subtypes. The duplicates were removed by using a FSC-area versus FSC-height/width plot. Viable and CD45+ cells were gated initially followed by gating CD14+ cells from the Lin(−) population. The HLA-DR(−) population was gated from CD14+ population defined as MDSC-1 (Lin^−^CD14^+^HLA-DR^−^). The MDSC-2 population (Lin^−^CD14^+^CD124^+^) comprising CD124+ cells were gated from the CD14+ cells by plotting CD124+ against it.

## Abbreviations

APC: allophycocyanin
aTregs: activated regulatory T cells
CTLA4: cytotoxic T-lymphocyte associated protein 4
Cy5.5: cyanine 5.5
Cy7: indocyanine 7
EMA: ethidium monoazide
FACS: fluorescence-activated cell sorting
FITC: fluorescein isothiocyanate
FoxP3: forkhead box P3
Her2: human epidermal growth factor receptor 2
IL: interleukin
IFN-γ: interferon gamma
Lin: lineage
MDSC: myeloid-derived suppressor cells
M1: matrix protein
NP: nucleoprotein
PBMCs: peripheral blood mononuclear cells
PD-1: programmed cell death protein 1
PDL-1: programmed death ligand 1
PE: phycoerythrin
PerCP: peridinin-chlorophyll protein
rTregs: resting regulatory T cells
TAAs: tumour-associated antigens
TNF: tumour necrosis factor
Tregs: regulatory T cells
