# Supplementary figures and images for: Presence of circulating Her2-reactive CD8 + T-cells is associated with lower frequencies of myeloid-derived suppressor cells and regulatory T cells, and better survival in older breast cancer patients
Source: Breast Cancer Res. 2015 Mar 10;17(1):34. doi: 10.1186/s13058-015-0541-z (PMC4377034; doi:10.1186/s13058-015-0541-z)

## Slide 1
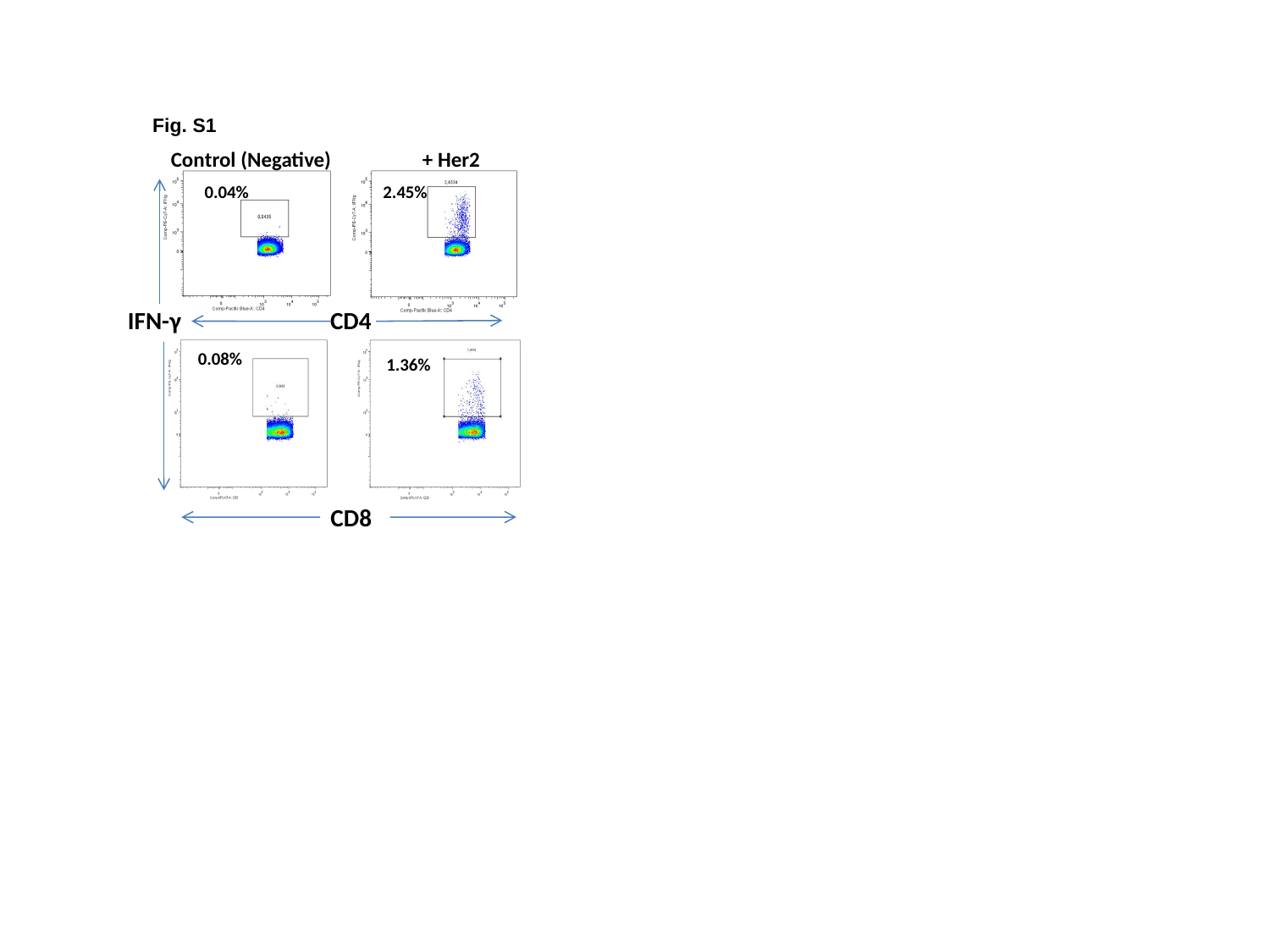

Fig. S1
Control (Negative)
+ Her2
0.04%
2.45%
	 CD4
IFN-γ
0.08%
1.36%
	 CD8

Supplement: Additional file 1: Figure S1. — CD4+ and CD8+ T-cell response to Her-2. The duplicates were removed by using a FSC-area versus FSC-height/width plot. The viable and CD3+ cells were gated earlier to plot CD4+ and CD8+ cells. A representative plot of control and Her-2 stimulated cytokine (for example: IFN-γ) producing cells for CD4+ T-cells and CD8+ T-cells are shown. [file 13058_2015_541_MOESM1_ESM.pptx]

## Slide 1
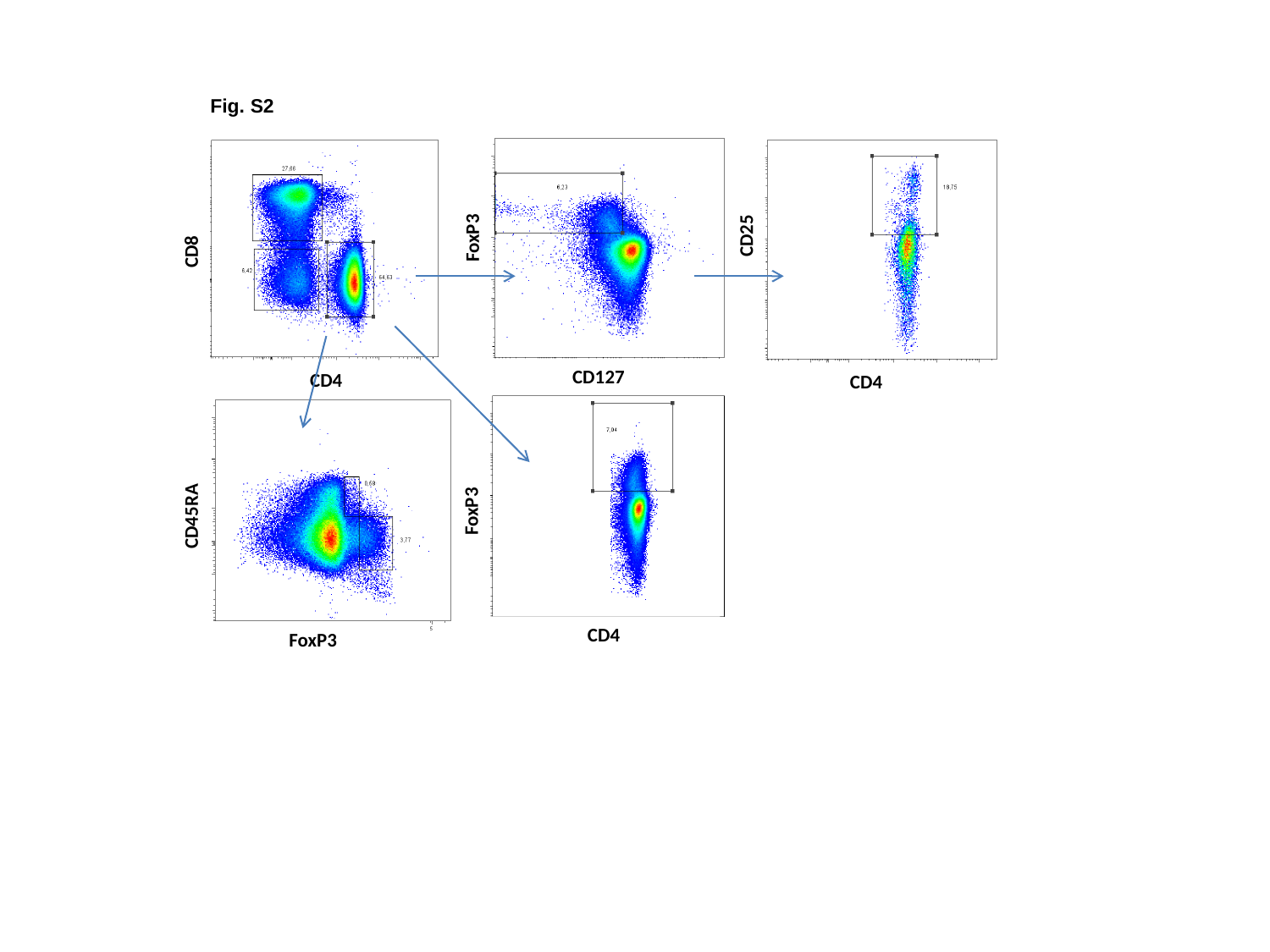

Fig. S2
CD25
FoxP3
CD8
CD127
CD4
CD4
FoxP3
CD45RA
CD4
FoxP3

Supplement: Additional file 2: Figure S2. — Gating strategy for regulatory T-cell subtypes. The duplicates were removed by using a FSC-area versus FSC-height/width plot. The viable and CD3+ cells were gated to plot CD4+ and CD8+ cells. FoxP3+ cells were gated from total CD4+ cells followed by gating of CD127lo and CD25+ cells. The activated Tregs (CD4+CD45−FoxP3hi) and resting Tregs (CD4+CD45RA+FoxP3+) were gated by plotting CD45RA against FoxP3+ cells. Also, FoxP3+ cells were gated from CD4+ T-cells (CD4 + FoxP3+). [file 13058_2015_541_MOESM2_ESM.pptx]

## Slide 1
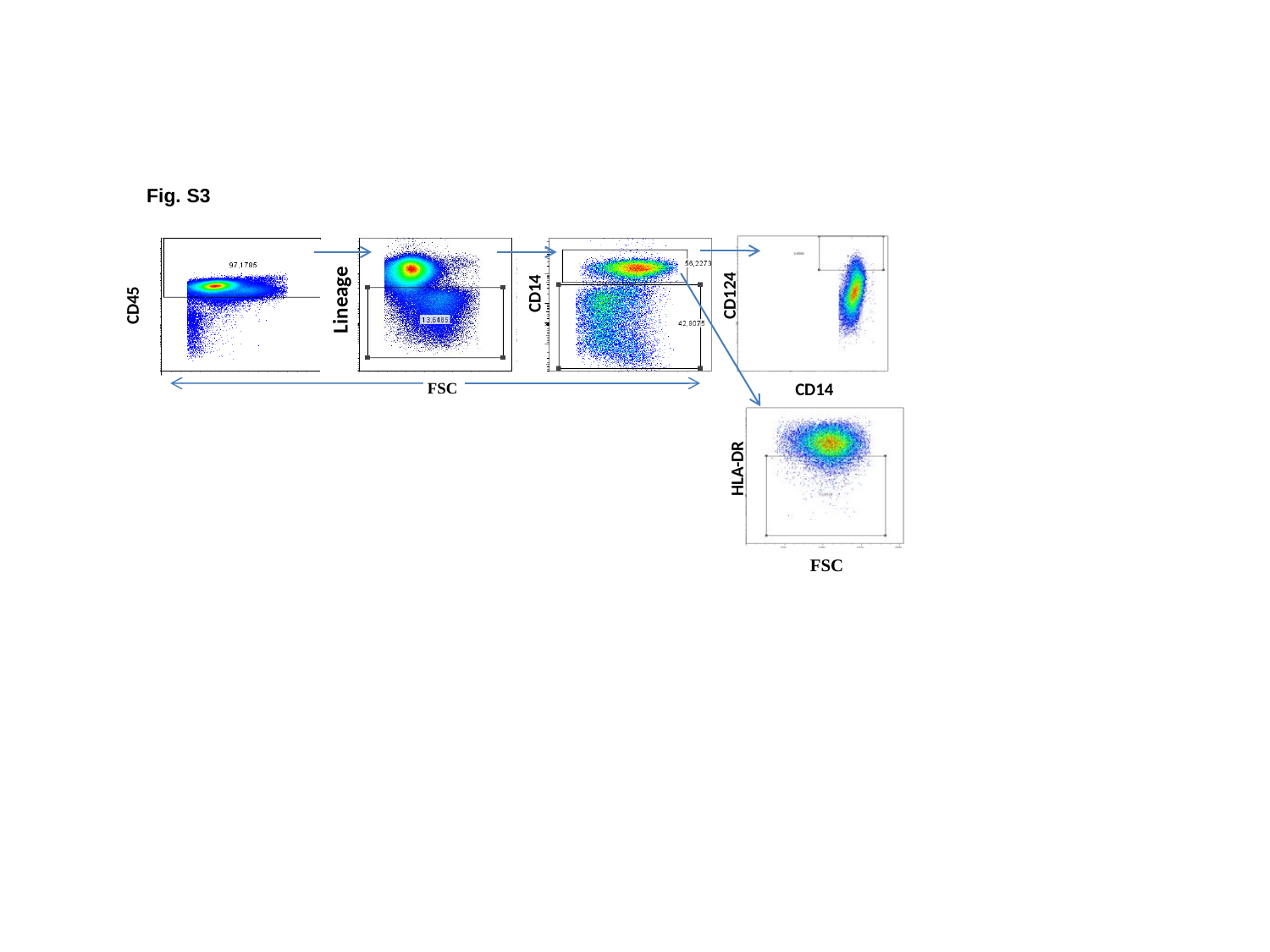

Fig. S3
 CD45
 Lineage
 CD14
 CD124
FSC
 CD14
 HLA-DR
 FSC

Supplement: Additional file 3: Figure S3. — Gating strategy for MDSC subtypes. The duplicates were removed by using a FSC-area versus FSC-height/width plot. Viable and CD45+ cells were gated initially followed by gating CD14+ cells from the Lin(−) population. The HLA-DR(−) population was gated from CD14+ population defined as MDSC-1 (Lin−CD14+HLA-DR−). The MDSC-2 population (Lin−CD14+CD124+) comprising CD124+ cells were gated from the CD14+ cells by plotting CD124+ against it. [file 13058_2015_541_MOESM3_ESM.pptx]
